# Deep Learning Algorithm for Tumor Segmentation and Discrimination of Clinically Significant Cancer in Patients with Prostate Cancer

**DOI:** 10.3390/curroncol30080528

**Published:** 2023-08-01

**Authors:** Sujin Hong, Seung Ho Kim, Byeongcheol Yoo, Joo Yeon Kim

**Affiliations:** 1 Department of Radiology, Inje University, College of Medicine, Haeundae Paik Hospital, Busan 48108, Republic of Korea; 2 Deepnoid Co., Ltd., Seoul 08376, Republic of Korea; 3 Department of Pathology, Inje University, College of Medicine, Haeundae Paik Hospital, Busan 48108, Republic of Korea

**Keywords:** magnetic resonance imaging (MRI), diffusion-weighted imaging (DWI), prostate cancer, Gleason score, deep learning

## Abstract

Background: We investigated the feasibility of a deep learning algorithm (DLA) based on apparent diffusion coefficient (ADC) maps for the segmentation and discrimination of clinically significant cancer (CSC, Gleason score ≥ 7) from non-CSC in patients with prostate cancer (PCa). Methods: Data from a total of 149 consecutive patients who had undergone 3T-MRI and been pathologically diagnosed with PCa were initially collected. The labelled data (148 images for GS6, 580 images for GS7) were applied for tumor segmentation using a convolutional neural network (CNN). For classification, 93 images for GS6 and 372 images for GS7 were used. For external validation, 22 consecutive patients from five different institutions (25 images for GS6, 70 images for GS7) representing different MR machines were recruited. Results: Regarding segmentation and classification, U-Net and DenseNet were used, respectively. The tumor Dice scores for internal and external validation were 0.822 and 0.7776, respectively. As for classification, the accuracies of internal and external validation were 73 and 75%, respectively. For external validation, diagnostic predictive values for CSC (sensitivity, specificity, positive predictive value and negative predictive value) were 84, 48, 82 and 52%, respectively. Conclusions: Tumor segmentation and discrimination of CSC from non-CSC is feasible using a DLA developed based on ADC maps (b2000) alone.

## 1. Introduction

Prostate cancer (PCa) is the second most frequently diagnosed cancer in men worldwide and the fifth most common cause of death [1]. Gleason score (GS) is a classification system based on the structure of PCa and is closely related to tumor aggressiveness. GS7 (particularly 3 + 4, International society of urological pathology (ISUP) grade 2) and above are classified as clinically significant cancers (CSCs) and GS6 (ISUP grade 1) as non-CSC [2].

PCa can be treated individually, depending on the degree of aggressiveness, risk of recurrence, and staging. Non-CSC is associated with relatively lower progression and mortality, suggesting a relatively good prognosis; thus, active surveillance and observation can be followed. However, as CSC is associated with a relatively high probability of adverse outcomes, active treatment, such as radical prostatectomy and/or radiation therapy, is required in general [3]. To date, the National Comprehensive Cancer Network (NCCN) guideline lists active surveillance for patients with favorable intermediate-risk prostate cancer (1 IR factor + Grade 1 or 2 + <50% positive biopsy cores) [4]. Another guideline promotes active surveillance for selected patients with low-volume GS 3 + 4 prostate cancer [5]. Therefore, efforts have been made to determine treatment policies based on risk stratification. However, due to the sampling errors inherent in systemic biopsy [6,7] as well as the possibility of complication associated with invasive approaches [8], interest in evaluating tumor aggressiveness using non-invasive imaging modalities such as magnetic resonance imaging (MRI) has increased.

There have been several promising studies on the usefulness of deep learning algorithms (DLAs), as based on mono-parametric or bi-parametric (bp) MRI for tumor detection of PCa [9,10,11,12,13,14,15]. DLA studies based on bp-MRI or mono-parametric MRI for segmentation and classification between CSC and non-CSC are less frequently found in the literature [2,3,9]. One of these studies undertook to distinguish CSC from non-CSC with deep-transfer-learning-based models using combined T2-weighted imaging (T2WI) and diffusion-weighted imaging (DWI) and a corresponding apparent diffusion coefficient (ADC) map, and the study revealed a similar diagnostic performance to that of prostate imaging reporting and data system (PIRADS) v.2.0 [3]. Both of those studies [2,3], however, employed sophisticated methods to combine the T2WI and DWI and used a low b value of 800 s/mm^2^. PIRADS score, moreover, has inherent limitations, such as a moderate inter-observer agreement and a probability scale by itself [16].

For PIRADS v.2.1, acquisition of high-b-value DWI (≥1400 s/mm^2^) is recommended. Furthermore, recent studies have shown that DWI b2000 is better than DWI b1000 for the localization of PCa [17,18]. However, to the best of our knowledge, DLA studies based on high-b-value DWI alone are scarce. Thus, we hypothesized that a DLA based on acquired DWI b2000 and corresponding ADC maps as a single input for discriminating CSC from non-CSC might deliver more beneficial results. The purpose of this study was to investigate the feasibility of using a DLA developed based on ADC maps (b2000) alone for tumor segmentation and discrimination of CSC from non-CSC in patients with PCa.

## 2. Materials and Methods

### 2.1. Patient Selection Criteria

**Figure 1:**
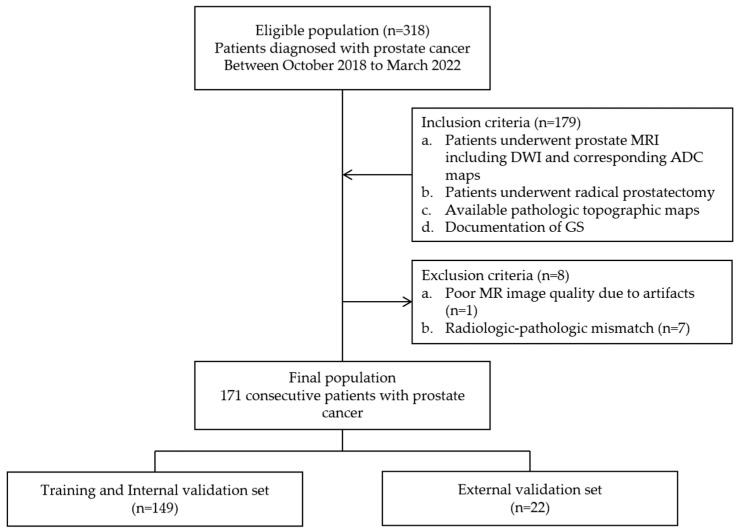
Flowchart of case enrollment process.

The pertinent institutional review board approved this retrospective study (IRB number blinded). Informed consent from patients was waived. Between October 2018 and March 2022, the relevant medical records of a total of 157 patients meeting the following inclusion criteria were collected: (i) complete 3T-MRI, including DWI and corresponding ADC maps, (ii) histological diagnosis of PCa and topographic map availability via radical prostatectomy and (iii) GS documentation availability via pathological reports. Among them, 8 patients were excluded based on one of the following exclusion criteria: (i) poor MR image quality due to severe artifacts (n = 1) or (ii) incomplete pathologic topographic map (n = 7). Finally, 149 patients (mean age: 69.2 years, range: 47–84 years) were enrolled for the training and internal validation datasets (80 and 20% of the data, respectively). For external validation, 22 consecutive patients (mean age: 69.6 years, range: 56–80 years), for whom five different MR machines had been employed and different parameters applied, were separately recruited during the same period. The case enrollment process is summarized in Figure 1.

### 2.2. MRI Technique

All of the MRI examinations for the training and internal validation datasets were performed using a 3.0-T MR machine (Achieva TX; Philips, Best, The Netherlands) with a parallel-array torso coil (SENSE Torso/cardiac coil; USA Instruments, Gainesville, FL, USA).

The scanning protocol was composed of axial, sagittal and coronal T2-weighted turbo spin-echo (TSE) and axial DWI sequences (b values, 0, 100, 1000, 2000 s/mm^2^). Corresponding ADC maps were generated for the designated b values, respectively. The detailed scan parameters are summarized in Table 1.

### 2.3. Data Processing

Two radiologists (with 18 and 3 years of experience, respectively) determined the tumor and whole-gland borders by consensus on axial ADC maps generated from b values of 0 and 2000. For segmentation, they reviewed T2WI in 3 planes and DWI (b = 2000 s/mm^2^) after referencing the topographic map as a ground truth. After determination of the tumor and gland borders, the junior radiologist drew the regions of interest (ROIs) along the determined tumor and gland borders on the ADC maps (b = 2000 s/mm^2^) using DEEP:LABEL software v.1.0.4 (Deepnoid, Seoul, Republic of Korea). When there were multiple tumors in a patient, the largest one was considered as the index tumor. The reviewers also recorded the PIRADS score for the index tumor based on PIRADS v2.1. The order of patients was random. The reviewers were blinded to the patients’ GS.

### 2.4. DL Architecture for Tumor and Gland Segmentation

As a convolutional neural network (CNN), U-Net was used for tumor and gland segmentation due to its high accuracy at various image sites. This architecture consists of a down-sampling encoder for features learning and an up-sampling decoder for feature production, and it is efficient, even with small datasets [19].

In the gland segmentation, each of the following pre-processing steps was performed for overall segmentation effectiveness. All of the labeled images were cropped with a margin of 5 pixels for delineation of the borders of the prostate gland. The Min–Max normalization guaranteed that all features were of the same scale. Finally, all of the images were resized to 128 × 128 pixels for use as inputs to the U-Net architecture for gland segmentation. Several hyper-parameters were tested to train the optimal DLA, for which purpose the Adam optimizer (learning rate: 0.001, decay rate: 0.95) was selected. In the tumor segmentation, the same pre-processing steps were performed, and the Adam optimizer (learning rate: 0.0001, decay rate: 0.95) was again employed for DLA training.

After tumor and gland segmentation, all of the labeled tumor data (148 images for GS6, 580 images for GS7) and gland data (535 images for GS6, 935 images for GS7) were used to evaluate the DLA predictive performance for accuracy, sensitivity, specificity, positive predictive value (PPV), negative predictive value (NPV) and Dice score.

### 2.5. DL Architecture for Tumor Classification

#### 2.5.1. Training Architecture

For tumor classification, the labeled tumor data were filtered with a cutoff of 25 pixels. Finally, 93 images for GS6 and 372 images for GS7 were used. For balanced training, the GS7 images were randomly allocated into four subsets of 93 images each in order to match the number of GS6 images. Therefore, 186 GS6/7 images were divided into 146 images for use as a training dataset and 40 for use as an internal validation dataset in each session. The Min–Max normalization and resizing steps were performed in the same manner as for the segmentation task.

Several CNNs, such as Inception, ResNet and DenseNet, were trained for tumor classification, and DenseNet 201 was selected for tumor classification due to its superior performance in distinguishing GS6 from GS7 [20,21,22]. DenseNet connects each layer to every other layer in a feed-forward manner. It also alleviates the vanishing-gradient problem, strengthens feature propagation, encourages feature reuse and substantially reduces the number of parameters [22]. It has shown good performance, even with an insufficient dataset. In the present study, based on four training and internal validation sessions, the DLA with the best diagnostic performance was selected and applied for external validation. All of the data processing as well as DL and training procedures were implemented in DEEPPHI (http://www.deepphi.ai/, accessed on 25 April 2022), a web-based open artificial intelligence platform.

#### 2.5.2. External Validation

For external validation of segmentation and classification, 22 consecutive patients from 5 different institutions (25 images for GS6, 70 images for GS7) representing different MR machines each with different parameters were recruited. The MR machines consisted of 1.5T (n = 1) and 3.0T (n = 21) scanners, and the images with the highest b values of DWI were composed of b800 (n = 1), b1000 (n = 4) and b2000 (n = 17). A total of 95 tumor slices (25 GS6 images, 70 GS7 images) and 180 gland slices were included and analyzed in order to externally validate the DLA that had been developed with the training dataset.

### 2.6. Reference Standard

Dedicated urologists performed the radical prostatectomies. A dedicated pathologist assessed each pathological slide according to the Gleason grading system [23] and drew up a topographic map that served as the ground truth for tumor segmentation on MRI. For classification of CSC and non-CSC, the GS, as obtained after surgery, was set as the gold standard. CSC was defined as GS ≥ 7 and non-CSC as GS6 [24].

### 2.7. Statistical Analysis

For the categorical data, the chi-square test or Fisher’s exact test was used to find any difference between the training and external validation datasets. For the continuous data, the *t*-test was used. The Dice score was used to quantify the performance of image segmentation. A Dice score of 1.0 means perfect overlap, and a score of 0.0 corresponds to no overlap [25]. The diagnostic performance for classification was calculated via receiver operating characteristic (ROC) curve analysis and expressed as the area under the ROC curve (AUC). Diagnostic predictive values, including accuracy, PPV and NPV, were also estimated under the maximal AUC. For all of the statistical calculations, MedCalc software for Windows (MedCalc Software version 20.111, Mariakerke, Belgium) was used. A *p* value of less than 0.05 was considered statistically significant.

## 3. Results

### 3.1. Patient Demographics

The age, prostate-specific antigen level, GS, PIRADS score and tumor location were not significantly different between the training and external validation datasets. The average time interval between MRI and surgery was 37.0 days (range, 5–447 days). The average volume of GS 6 tumors was not significantly different from that of GS 7 tumors in both training and internal validation sets (GS 6, 4.1 ± 6.6 cm^3^; GS 7, 7.0 ± 7.3 cm^3^, *p* = 0.1822) and the external validation set (GS 6, 1.9 ± 1.9 cm^3^; GS 7, 6.3 ± 6.1 cm^3^, *p* = 0.1348). The patients’ demographic data and analysis results are presented in Table 2.

### 3.2. Diagnostic Performance of DLA

**Figure 2:**
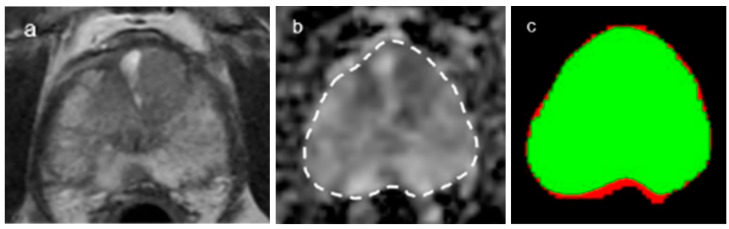
A representative case of gland segmentation. (**a**,**b**) The Dice score for the gland segmentations was 0.94. Axial T2-weighted image (**a**) and corresponding ADC map (**b**) (b = 2000 s/mm^2^) with gland segmentation ((**b**), dotted lines). (**c**) Segmentation through the convolutional neural network (CNN, U-Net) shows that the green color represents the matched area and the red color the unmatched area.

**Figure 3:**
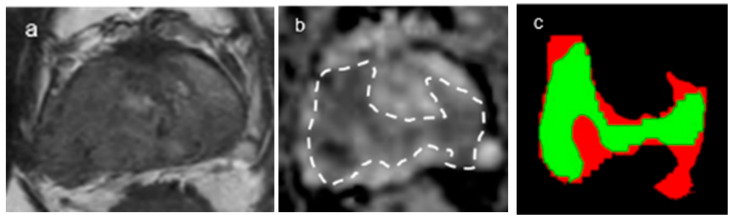
A representative case of tumor segmentation with GS7(4 + 3). (**a**,**b**) The Dice score for the tumor segmentations was 0.78. Axial T2-weighted image (**a**) and corresponding ADC map (**b**) (b = 2000 s/mm^2^) with tumor segmentation ((**b**), dotted lines). (**c**) Segmentation through the convolutional neural network (CNN, U-Net) shows that the green color represents the matched area and the red color the unmatched area.

In terms of gland segmentation, U-Net had a sensitivity of 95%, a specificity of 96% and a Dice score of 0.951 for internal validation and 92%, 97% and 0.9413, respectively, for external validation (Figure 2). As for tumor segmentation, it had a sensitivity of 82%, a specificity of 96% and a Dice score of 0.822 for internal validation and 77%, 95% and 0.7776, respectively, for external validation (Figure 3) (Table 3).

**Figure 4:**
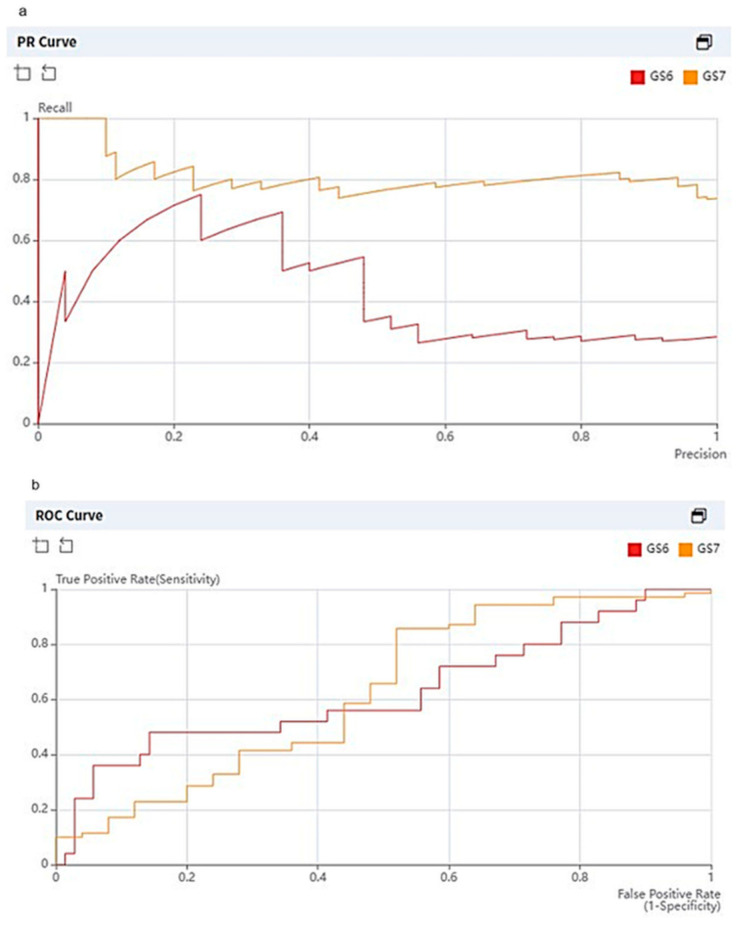
Graphs showing precision recall curve (**a**) and receiver operating characteristic (ROC) curve (**b**) of deep learning algorithm (DLA) for tumor classification as applied to external validation dataset. Average precision for GS6 and GS7 was 0.4462 and 0.8149, respectively. The DenseNet 201 classifier achieved an AUC of 0.6269 for both GS6 and GS7.

**Figure 5:**
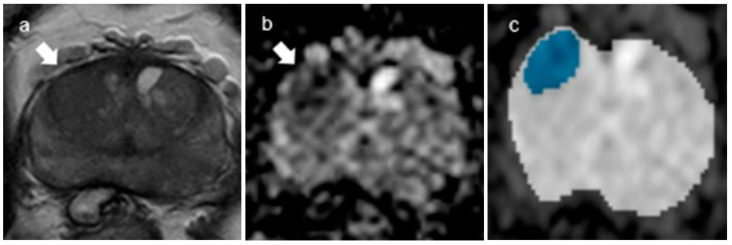
A representative case of misclassification: over-estimation of GS6 as GS7. (**a**,**b**) Axial T2-weighted image (**a**) and ADC map (**b**) (b = 2000 s/mm^2^) show a tumor in the Rt. mid-transitional zone (arrows). (**c**) Segmentation and classification through the convolutional neural network (CNN, DenseNet 201) show the tumor area as blue color.

**Figure 6:**
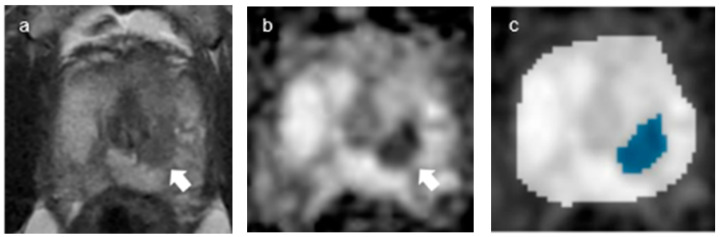
A representative case of misclassification: under-estimation of GS7 as GS6. (**a**,**b**) Axial T2-weighted image (**a**) and ADC map (**b**) (b = 2000 s/mm^2^) show a tumor in the Lt. mid-transitional zone (arrows), respectively. (**c**). Segmentation and classification through the convolutional neural network (CNN, DenseNet 201) show the tumor area as blue color.

As for classification, the overall accuracies of internal and external validation were 73 and 75%, respectively. For internal validation, the diagnostic predictive values for CSC (hereafter sensitivity, specificity, PPV and NPV, in order) were calculated as 72, 74, 74 and 72%, respectively. For external validation, the diagnostic predictive values were estimated as 84, 48, 82 and 52%, respectively (Table 4). The DenseNet 201 classifier achieved an AUC of 0.6269. The average precision scores for GS6 and GS7 were 0.4462 and 0.8149, respectively (Figure 4). Out of a total of 95 tumor slices (25 GS6 images, 70 GS7 images), 13 slices of GS6 were over-estimated as GS7 and 11 slices of GS7 were under-estimated as GS6 (Figure 5 and Figure 6).

## 4. Discussion

Regarding tumor segmentation, the DLA, which was based on ADC maps (b2000) alone in our study, showed Dice scores of 0.94 and 0.78 for gland and tumor segmentation, respectively. Our observations are similar to those of a previous study on mono-parametric MRI. Alkadi et al. reported that the accuracy of a DLA, which was based on T2WI only for tumor segmentation, was 89% [9]. As for tumor segmentation based on bp-MRI, Schelb et al. reported that the Dice scores for a DLA based on bp-MRI (T2WI + DWI b1500) using U-Net for detection and segmentation of CSCs were 0.35 for tumors and 0.89 for glands [10]. Relative to this latter study, in our opinion, the relatively high Dice score for tumor segmentation in this present study might have been due to the use of DWI b2000. Rosenkrantz et al. revealed that DWI b2000 achieved significantly higher sensitivity for tumor detection than b1000 [17]. Vural et al. found that b2000 showed the best lesion conspicuity and background suppression among b values of 1500, 2000 and 3000 [26]. In addition, Cha et al. reported that the optimal b value of DWI was within a range of 1700–1900 for the detection of a prostatic lesion [27].

In terms of tumor classification, the DLA in the present study showed an accuracy of 75% and an AUC of 0.63 in external validation. Recently, many deep-learning-based computer-aided detection/classification (DL-CADe/CADx) systems have been developed to assist human radiologists. Rampun et al. compared the 11 different CAD systems employed to detect peripheral-zone cancer (GS ≥ 7), only for T2WI on 3T-MRI [12]. The results varied from an AUC of 0.69 (k-Nearest Neighbor classifier) to 0.93 (combined Bayesian Network and Multilayer Perceptron classifiers), according to the applied CNNs. Ishioka et al. reported AUCs ranging from 0.636 to 0.645 for tumor (GS ≥ 6) detection via combined U-Net with ResNet50, as trained on T2WI only with the 1.5T-MRI machine [13]. Although only ADC maps (b2000) were used in our study, the diagnostic performance for tumor classification seems comparable to mono-parametric MRI using T2WI alone.

Beyond mono-parametric MRI, Arif et al. found that a DLA (Keras with TensorFlow) developed based on bp-MRI (T2WI + DWI b800) showed an AUC of 0.89, a sensitivity of 94% and a specificity of 74% for discrimination of CSCs from non-CSCs [2]. In our study, the sensitivity and specificity for GS7 were 84 and 48%, respectively. The relatively low specificity might have been due to the mono-parametric MRI based study, without any other sequences. Zhong et al. compared the diagnostic performance of DLA models trained with T2WI (DLAT2) alone, ADC images (DLAADC) alone and combined T2WI and ADC images (DLAT2 + ADC) in discriminating CSC from non-CSC [3]. All three models showed the same sensitivity of 77%, and the combined T2WI and ADC (b800) information, notably, helped to reduce false-positive prediction, thereby improving the specificity from 52 to 64% after adding DLAT2 + ADC to DLAADC.

Considering the previously mentioned merits of DenseNet, including reduction in the vanishing gradient, enhancement of feature propagation, reuse of features, reduction in the number of parameters [22] and its robustness, we think that our DLA, as developed by DenseNet and based on ADC maps, could be a simple and convenient option for the differentiation of CSC from non-CSC.

Our study has several limitations. First, tumor segmentation was conducted not on a three-dimensional (3D)-volume data basis but on a 2D-image basis, due to the inherent technical limitation of the segmentation tool. Therefore, when a classification error occurred in one tumor-bearing slice, there was a tendency that those errors would continue to consecutive slices. As a result, diagnostic performance for tumor classification might have been underestimated. Second, there is a possibility of selection bias, as only GS7 tumors were included in the CSC group. However, GS8-or-higher tumors are frequently advanced cases of metastatic disease, for which systemic chemotherapy would be adopted rather than radical prostatectomy. Considering the purpose of this study, to separate the group capable of surveillance from the group that is not, the study was conducted except for tumors with a score of GS8 or higher that were already inoperable. It would be better to have a larger sample size for GS6 in the external validation set; however, it was difficult to enroll patients with GS6. Patients with GS6 have a relatively good prognosis; thus, active surveillance and observation can be followed instead of radical prostatectomy. Third, the DLA’s value added to the human radiologists’ performance for tumor classification was not investigated. As for the added value, several previous observations have been reported in the literature [14,15]. Winkel et al. reported that the DL-CAD system increased the diagnostic accuracy in detecting clinically suspicious lesions (PIRADS ≥ 4) and reduced both the inter-reader variability and the reading time [14]. However, it was beyond the scope and aim of the present study. To investigate the added value of a DLA to the performance of human radiologists for tumor classification, further studies on DLA efficacy in this regard are warranted.

## 5. Conclusions

In conclusion, tumor segmentation and classification of PCa through a DLA developed based on ADC maps (b2000) alone are feasible.

## Figures and Tables

**Table 1:** MRI sequence parameters for training set.

Parameters	T2-Weighted Axial, Sagittal, and Coronal TSE	DWI (b = 0, 100, 1000 and 2000 s/mm^2^)
TR (msec)	3370.7	5725
TE (msec)	100	77.8
Slice thickness (mm)	3	3
Slice gap (mm)	0.3	0.3
Matrix size	316 × 272	120 × 118
NEX	1	1
FOV (mm × mm)	220 × 220	240 × 240
Number of slices	30	30

TR, repetition time; TE, echo time; NEX, umber of excitations; FOV, Field of view; TSE, Turbo spin echo. Note that diffusion-weighted imaging (DWI) was performed using the single-shot echo-planar imaging (SS-EPI) technique.

**Table 2:** Demographic data and analysis results for study population.

Parameter	All	Training and Internal Validation Sets (n = 149)	External Validation Set (n = 22)	*p* Value
Mean Age, years [range]	69.2982 [47–84]	69.2483 [47–84]	69.6364 [56–80]	0.8049
Mean PSA, ng/mL [range]	14.6315 [0.85–149]	14.4478 [0.85–149]	21.1709 [3.0–131]	0.3597
GS, n (%)				
6	46 (27)	40 (27)	6 (27)	0.9307
7	125 (73)	109 (73)	16 (73)	0.9912
3 + 4	89	76	13	
4 + 3	36	33	3	
PIRADS v2.1, n (%)				
3	17 (10)	17 (11)	0 (0)	0.1131
4	55 (32)	49 (33)	6 (27)	0.7006
5	99 (58)	83 (56)	16 (73)	0.3307
Tumor location, n (%)				
Peripheral zone	92 (54)	81 (54)	11 (50)	0.8245
Transitional zone	48 (28)	38 (26)	10 (45)	0.1204
Fibromuscular zone	4 (2)	4 (3)	0 (0)	0.4422
Diffuse	27 (16)	26 (17)	1 (5)	0.1453

GS, Gleason score; PSA, prostate-specific antigen.

**Table 3:** Diagnostic predictive values of DLA for segmentation of glands and tumors.

	Accuracy (%)	Sensitivity (%)	Specificity (%)	PPV (%)	NPV (%)	Dice Score
Gland						
Internal validation	96	95	96	95	96	0.951
External validation	95	92	97	96	93	0.9413
Tumor						
Internal validation	93	82	96	83	96	0.822
External validation	92	77	95	79	95	0.7776

U-Net was used for deep learning algorithm (DLA).

**Table 4:** Diagnostic predictive values of DLA for tumor classification.

	Accuracy (%)	Sensitivity (%)	Specificity (%)	PPV (%)	NPV (%)	AUC
Internal validation set						
CSC	73	72	74	74	72	
External validation set						
CSC	75	84	48	82	52	0.6269

DenseNet 201 was used for the deep learning algorithm (DLA). CSC, clinically significant cancer.

## Data Availability

The data presented in this study are available on request from the corresponding author. The data are not publicly available due to patients’ privacy.
